# Clinical course of neonatal acute kidney injury: multi-center prospective cohort study

**DOI:** 10.1186/s12887-022-03200-w

**Published:** 2022-03-15

**Authors:** O. C. Pantoja-Gómez, S. Realpe, Ginna Cabra-Bautista, J. M. Restrepo, O. L. Prado, A. M. Velasco, G. E. Martínez, S. Leal, A. Vallejo, Jose Andrés Calvache

**Affiliations:** 1 Departamento de Pediatría, Universidad del Cauca, Popayán, Colombia; 2 Hospital Susana López de Valencia, Popayán, Colombia; 3 Servicio de Nefrología Pediátrica, Fundación Valle del Lili, Cali, Colombia; 4 Hospital Universitario San Jose, Popayán, Colombia; 5 Departamento de Anestesiología, Universidad del Cauca, Popayán, Colombia; 6 Department of Anesthesiology, Erasmus University Medical Center, Rotterdam, The Netherlands

**Keywords:** Acute kidney injury, Infant, Newborn, Renal replacement therapy, Mortality, Intensive care units, Neonatal, KDIGO

## Abstract

**Background:**

Neonatal acute kidney injury (AKI) has been associated with unfavorable outcomes, including increased mortality. We aimed to describe the clinical course and outcomes during the first 7 days after diagnosis in newborns with AKI in three neonatal intensive care units in Popayán-Colombia.

**Methods:**

Multi-center prospective cohort study conducted between June 2019 and December 2020 in three NICUs after ethical approval. We included newborns between 2 and 28 days of life, first diagnosed with AKI using the KDIGO classification modified for newborns which consider increased serum creatinine values over baseline values as well as urine output over time in hours or both. Patients with chromosomal abnormalities, major kidney malformations, and complex congenital heart disease were excluded. Patients were followed for up to 7 days after diagnosis and the maximum KDIGO stage, recovery of kidney function, need for renal replacement therapy and cumulative incidence of death were evaluated.

**Results:**

Over the 18 months of the study, 4132 newborns were admitted to the NICUs, and 93 patients (2.25, 95% CI 1.82–2.75%) developed neonatal AKI. 59.1% of the newborns were premature and there were no differences in severity according to gestational age. During follow-up, the maximum KDIGO was 64.5% for AKI-stage 1, 11.8% for AKI-stage 2, and 23.7% for AKI-stage 3. Kidney function recovery was higher in AKI-stage 1 patients vs. AKI-severe (AKI-stage 2 and 3) (95% vs. 48.5%). Five patients (5.4%) received renal replacement therapy and 15 died (16.1%), four in AKI-stage 1 vs. 11 in AKI-severe (6.7% vs 33.3%).

**Conclusions:**

Newborns admitted to the NICUs can develop AKI regardless of gestational age, and it is more frequent between the second and ninth days of life. More patients whit AKI-stage 1 recover and die less than those in a severe stage.

## Background

Acute kidney injury (AKI) is defined as the drop or sudden loss of kidney function and is characterized by accumulation of waste products, loss of fluid and electrolyte balance, and alteration of acid-base homeostasis [1–3]. It is a common condition in critically ill newborns hospitalized in neonatal intensive care units (NICUs), and it is considered an independent risk factor that increases mortality, length of stay, and health care costs [4–8].

The reported incidence of neonatal AKI varied due to the use of different diagnostic criteria in newborns, particularly difficult to use in premature newborns [9–13]. The diagnostic and classification criteria of the Kidney Disease: Improving Global Outcomes (KDIGO) modified for newborns are currently available, based on quantitative changes in serum creatinine levels and/or a drop in urine output, and their use has provided a better approach to the diagnosis of neonatal AKI [14].

Recognizing neonatal AKI as an independent mortality risk factor, in 2013 the International Society of Nephrology published the initiative of zero preventable deaths from AKI by 2025 ‘0by25’. This strategy attaches great importance to the identification of risk factors for AKI to enable early diagnosis and intervention [15, 16].

Despite advances in the study of neonatal AKI, the early course is unclear, especially during the first few days after diagnosis, which determine progression to acute kidney disease (decrease of kidney function between seven and ninety days) [17]. We aimed to describe the clinical course and outcomes in newborns diagnosed with AKI during the first 7 days after diagnosis in three NICUs in Popayán-Colombia.

## Methods

A multi-center prospective observational cohort study was conducted between June 27, 2019, and December 26, 2020, in three NICUs, namely, San Jose University Hospital (HUSJ), Susana López de Valencia Hospital (HSLV), and La Estancia Clinic (CLE) with a total capacity of 60 beds in the 3 NICUs, located in the city of Popayan, department of Cauca in southwestern Colombia, South America.

All newborns between 2 and 28 days of life admitted to the NICUs, first diagnosed with AKI during their hospital stay, were included and followed for 7 consecutive days. Patients with chromosomal abnormalities, malformations incompatible with life, complex congenital heart disease (tetralogy of Fallot, hypoplastic left heart syndrome, transposition of the great vessels, total anomalous pulmonary venous return, ectopia cordis) and major kidney malformations (Bilateral Agenesis and Bilateral Kidney Hypoplasia-Dysplasia) were excluded because of their association with AKI. Unilateral kidney malformations were included because the contralateral kidney can supply kidney function and because congenital kidney and urinary tract abnormalities eventually cause decreased kidney function but not in the neonatal period.

Neonatal AKI was defined and diagnosed with KDIGO classification modified for newborns which considers increased serum creatinine values over baseline values as well as urine output over time in hours (Table 1) [14]. Patients in AKI-stage 2 and AKI-stage 3 were classified as AKI-severe [18]. Maximum KDIGO was defined after 7 days of follow-up in patients with the highest serum creatinine value or the lowest urine output and their corresponding classification.

**Table 1:** Neonatal AKI KDIGO Classification

Stage	SCr	Urine Output
1	SCr increase ≥0.3 mg/dL within 48 h or SCr increase ≥1.5–1.9 x reference SCr^a^ within 7 days	< 0.5 mL/kg/h for 6–12 h
2	SCr increase ≥2 to 2.9 x reference SCr^a^	< 0.5 mL/kg/h for ≥12 h
3	SCr increase ≥3 x reference SCr^a^ or SCr ≥ 2.5 mg/dL or Receipt of dialysis	< 0.3 mL/kg/h for ≥24 h or anuria for ≥12 h

*SCr* Serum creatinine. ^a^Baseline SCr is defined as the lowest previous SCr value

Adapted from Jetton, J. G., & Askenazi, D. J. (2014). *Acute Kidney Injury in the Neonate. Clinics in Perinatology, 41* (3)*, 487–502*

Patients hospitalized in the NICUs were managed with an individualized approach in each unit. Serum creatinine measurements were made by the indication for each patient. Urine output was measured directly in all infants with urinary catheters and indirectly using diaper weight in those without a urinary catheter. In the three NICUs, diapers were weighed in calibrated electronic scales, subtracting the weight of the dry diaper (20 g for term infants and 10 g for preterm infants), and the result was used to calculate urine output according to the respective time schedule.

The first day of neonatal AKI diagnosis was designated as day 1, after which daily follow-up was performed for 7 days to assess the course of the disease in terms of recovery (reduction of serum creatinine to baseline levels for gestational age or improvement of urine output), need for renal replacement therapy (RRT) in case of anuria or a glomerular filtration rate (GFR) < 20 ml/min/1.73, and death. Assistant researchers completed the data collection tools designed for the study.

Newborn data collected included sociodemographic characteristics: sex, gestational age on admission, birth weight, ethnicity, maternal age, type of delivery, area of residence, and social security status; gestational history: intrauterine growth restriction (IUGR), completed prenatal visits according to gestational age [19], chorioamnionitis, urinary tract infections, sepsis, minor kidney malformations, prenatal medications, i.e., antihypertensives, antibiotics or steroids; comorbidities during NICUs hospitalization: perinatal asphyxia, sepsis, patent ductus arteriosus, necrotizing enterocolitis, respiratory distress syndrome, pneumonia, neonatal respiratory distress syndrome, shock, electrolyte imbalance, metabolic acidosis; and interventions: mechanical ventilation, central catheterization, total parenteral nutrition, blood products transfusion, use of inotropes or nephrotoxic agents.

The primary outcomes of the study were kidney function recovery, the need for RRT, and cumulative incidence of death over the 7-day follow-up period in the NICUs. The secondary outcomes were the cumulative incidence of overall neonatal AKI and AKI-severe, besides the determination of the maximum AKI stage (maximum KDIGO) observed during follow-up.

### Statistical analysis

Initially, a descriptive analysis of sociodemographic and clinical admission data was performed. Values are presented as means and standard deviations or medians and inter-quartile ranges. Categorical variables are described using absolute frequencies and proportions.

This study was designed to estimate kidney function recovery, the need for RRT, and mortality in the patients admitted to each of the NICUs with a diagnosis of AKI. Cumulative incidence and confidence interval (95% CI) of neonatal AKI were calculated in critically ill newborns admitted to the NICUs during the study period. Patients with AKI stages 1, 2, and 3 on the first day of diagnosis and maximum KDIGO are reported as frequencies and proportions.

Results after 7 days of follow-up are presented in terms of kidney function recovery, RRT, and mortality, stratified by severity as AKI-stage 1 and AKI-severe. They were compared using the X^2^ test, and statistical significance was predefined using a *p* value < 0.05. Analyses were performed using R [20].

## Results

**Fig. 1:**
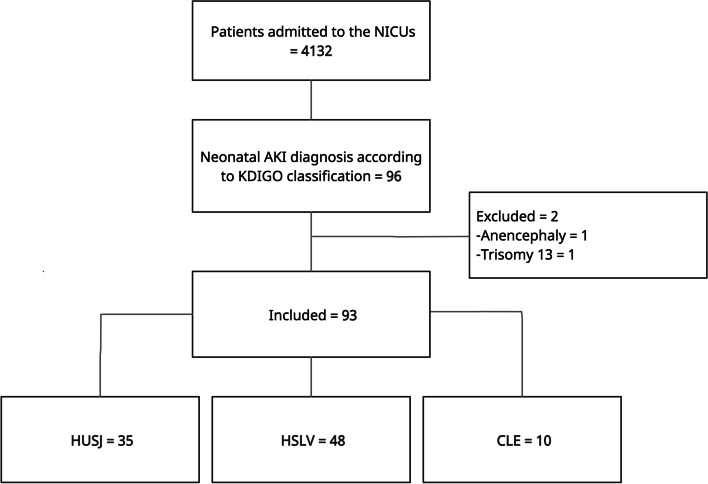
Flow chart of patient inclusion in the study

During the 18-month recruitment period, 4132 newborns were admitted to the NICUs of the participating hospitals (HUSJ = 1019, HSLV = 1872, CLE = 1241). Of them, 93 developed AKI during their stay in the NICUs (HUSJ = 35, HSLV = 48, CLE = 10), with a cumulative incidence of 2.25% (95% CI 1.82–2.75%) (Fig. 1).

Of the patients diagnosed with neonatal AKI included in the cohort, 40% were term newborns, and the majority were of mestizo ethnicity and belonged to low socioeconomic status (Table 2).

**Table 2:** Sociodemographic characteristics of patients with AKI (*n* = 93)

Variables	*n* (%)
Male sex	49 (52.7)
Gestational age on admission, Me (IQR)	35 (29–39)
22 to 28 weeks	21 (22.6)
29 to 36 weeks	34 (36.6)
> 36 weeks	38 (40.9)
Birthweight, Me (IQR)	1900 (1140–2970)
< 1500 g	36 (38.7)
1500 a 2500 g	20 (21.5)
> 2500 g	37 (39.8)
Ethnicity	
Mestizo	74 (79.6)
Indigenous	14 (15.1)
Afro-Colombian	5 (5.4)
Maternal age (years), Me (IQR)	25 (19–33)
Cesarean delivery	60 (64.5)
Rural area residence	52 (56)
Socioeconomic status	
1	76 (81.7)
2	12 (12.9)
3	4 (4.3)
4	1 (1.1)

*Abbreviations*: *Me* median, *IQR* inter-quartile range, *g* grams

In terms of maternal characteristics, 87% had incomplete prenatal follow-up for gestational age at the time of birth. Twenty percent had a history of IUGR, some form of infection during gestation, and had received antihypertensives, antibiotics, or steroids prenatally. The most frequent comorbidities were sepsis and respiratory distress syndrome. Likewise, between 80 and 90% of patients required the use of mechanical ventilation, central lines, total parenteral nutrition, inotropes, or nephrotoxic medicine (Table 3).

**Table 3:** Maternal and clinical characteristics of patients with AKI (*n* = 93)

Variables	*n* (%)
**Gestational history**	
Intrauterine growth restriction	19 (20.4)
Chorioamnionitis, urinary tract infection, or sepsis	22 (23.7)
Prenatal kidney malformation	8 (8.6)
Prenatal antihypertensive medications	19 (20.4)
Prenatal antibiotics	22 (23.7)
Prenatal steroids	22 (23.7)
Incomplete prenatal care	81 (87.1)
**Clinical conditions and comorbidities in the newborns**	
Sepsis	87 (93.5)
Respiratory distress syndrome	77 (82.8)
Patent ductus arteriosus	73 (78.5)
Pneumonia	66 (71)
Perinatal asphyxia	61 (65.6)
Shock	48 (51.6)
Electrolyte imbalance	46 (49.5)
Neonatal respiratory distress syndrome	31 (33.3)
Metabolic acidosis	29 (31.2)
Necrotizing enterocolitis	8 (8.6)
**Clinical interventions in the NICUs**	
Mechanical ventilation	74 (79.6)
Central catheterization (including umbilical)	79 (84.9)
Total parenteral nutrition	73 (78.5)
Nephrotoxic medicine	86 (92.5)
Inotropes	75 (80.6)
Transfusion of blood products	54 (58.1)

*Abbreviations*: *NICUs* Neonatal intensive care units

**Fig. 2:**
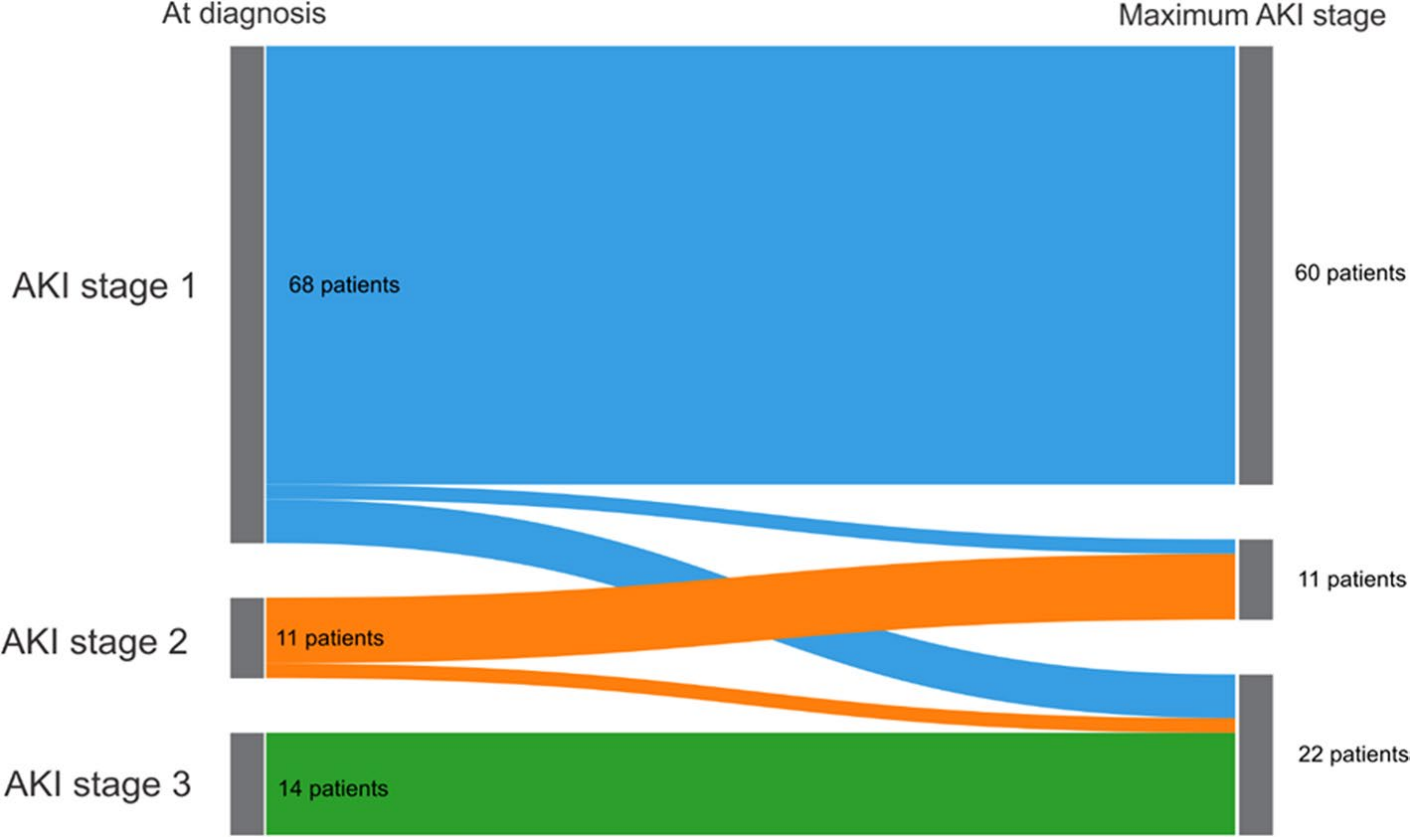
Progression of AKI diagnosis after 7 days of follow-up in the NICUs

The diagnosis of neonatal AKI was based on serum creatinine elevation in 48% of patients (51.6%), reduced urine output in 39 (41.9%), and both in 6 patients (6.5%). The average number of creatinine measurements per patient during the 7 days of follow-up was 2.8 (DE = 1.5). Of the 93 patients, 68 (73.1%) were AKI-stage 1, 11 (11.8%) AKI-stage 2 and 14 (15.1%) AKI-stage 3 on the first day of diagnosis. During follow-up, based on the maximum KDIGO reached, 60 patients (64.5%) were classified as AKI-stage 1, 11 (11.8%) as AKI-stage 2, and 22 (23.7%) as AKI-stage 3 (Fig. 2). Finally, a cumulative incidence of 0.8% (95% CI 0.55–1.11%) was found for AKI-severe after 7 days of follow-up.

All the newborns developed AKI between days 2 and 26 of neonatal life (median: 6 days, IQR: 2–9 days). Table 4 shows the distribution of patients diagnosed as AKI-stage 1 vs. AKI-severe by gestational age and birth weight.

**Table 4:** AKI-stage 1 vs. AKI-severe according to gestational age and birthweight

	Total Cohort *n* = 93 (%)	AKI stage 1 *n* = 60 (%)	AKI Severe *n* = 33 (%)
**Gestational age in weeks**			
22 to 28	21 (22.6)	11 (18.3)	10 (30.3)
29 to 36	34 (36.6)	26 (43.3)	8 (24.2)
> 36	38 (40.9)	23 (38.3)	15 (45.5)
**Birthweight**			
< 1500 g	36 (38.7)	22 (36.7)	14 (42.4)
1500 to 2500 g	20 (21.5)	14 (23.3)	6 (18.2)
> 2500 g	37 (39.8)	24 (40)	13 (39.4)

*Abbreviation*: *AKI* Acute kidney Injury

### AKI severity and outcomes

After 7 days of follow-up, 73 patients (78.5%) had recovered kidney function, with kidney function recovery being higher in patients with AKI-stage 1 vs. AKI-severe (95% vs. 48.5%, *P* < 0.000). Of the 93 patients with neonatal AKI, 5 (5.4%; 95% CI 0.18–12.1) received RRT during follow-up; peritoneal dialysis was the most commonly used modality in four patients, while hemodialysis in the form of continuous venovenous hemodiafiltration was used in one patient. Fifteen patients (16.1%; 95% CI 9.3–25.2) diagnosed with AKI died during their stay in the NICUs, four in AKI-stage 1 and 11 in AKI-severe (6.7% vs. 33.3%, *P* < 0.001).

## Discussion

This multi-center prospective cohort study describes the clinical course of neonatal AKI in critically ill patients in three NICUs, as well as outcomes in terms of kidney function recovery, need for RRT, and mortality at 7 days after diagnosis. This is one of the largest studies of neonatal AKI encompassing resource-limited settings with limited access to care. The study population included newborns of mestizo, indigenous and Afro-Colombian ethnicity, the majority from low socioeconomic status and with incomplete prenatal care for gestational age [19]. The latter two factors are related to barriers to healthcare access in pregnant women of our population and have not been reported in studies carried out in developed countries. Comorbidities found and treatments used were similar to the reported by other related studies [1, 5, 11, 21, 22].

A cumulative incidence of 2.25% of neonatal AKI in 7 days of follow-up was found, which is low when compared to those reported by Naunova (6.5%), Jetton (30%), and Shalaby (56%) [1, 5, 23]. Differences found in the incidence of the disease might be explained by the type of classification and eligibility criteria used for the diagnosis of AKI.

In our study, patients diagnosed with neonatal AKI developed the condition early on, the median being in the first 2–9 days of life. In 42% of cases, the diagnosis was based on urine output of less than 0.5 ml/kg/h as the only criterion. This finding underscores the importance of urine output as a diagnostic method for AKI in the NICUs. This information is in contrast with reports from developed countries, where the diagnosis was based on an increase of serum creatinine in more than 90% of patients [5, 23]. A 3:2 ratio was found between preterm and term newborns with AKI, with no differences in severity according to gestational age. These data are similar to those found in the AWAKEN study (Assessment of Worldwide Acute Kidney Injury Epidemiology in Neonates), although with a higher frequency of AKI in the extremes (22 to 29 weeks and > 36 weeks) [5].

In terms of severity, we found a higher frequency of AKI-stage 1, similar to other reports. However, the percentage of AKI-severe (35.5%) was lower than in other studies (45 and 53%) [5, 23]. When maximum KDIGO was used to assess progression during the 7 days of follow-up, it was found that one out of every 10 newborns worsened despite management received in the NICUs. Moreover, AKI-severe is associated with higher mortality [5, 22], pointing to the importance of early diagnosis and active management of AKI-stage 1 to achieve a kidney protective environment (hemodynamic stability, avoidance of hypoxia and nephrotoxic drugs, medication dose adjustments according to GFR, and adequate nutrition) to avoid progression and to determine the need to initiate RRT if there is no improvement.

Most of the patients with AKI-stage 1 recovered the kidney function over the 7 days of follow-up as compared to those with AKI-severe, of whom only close to one-half improved. These findings are relevant because one out of every five patients did not recover during follow-up and progressed to acute kidney disease [17]. These results are similar to those reported by Shalaby, who found persistent kidney impairment during the first month of life in newborns diagnosed with AKI and described that 27% were discharged with abnormal creatinine values [23].

Of the patients with neonatal AKI followed in this study, 5.4% required RRT, mainly in the form of peritoneal dialysis, considered as the modality of choice during the neonatal period because of ease of implementation and no need for vascular access or anticoagulation. Our findings are slightly higher than those reported by Jetton (4.1%) and Shalaby (1.7%), who also included newborns with correction of congenital heart disease in whom RRT is used more frequently [5, 23].

Our study found that 16.1% of patients died during follow-up. This result was higher than the 9.7% reported in the AWAKEN study [5] and lower than the 28.3% described by Shalaby [20] and the 32–70% reported in studies with premature newborns [1, 11, 21]. In general, follow-up in those studies continued during the entire stay in the NICU, as compared to the 7-day follow-up period of our study, corresponding to the AKI definition time. Only one of the three NICUs included in this study has access to therapeutic hypothermia for patients with hypoxic-ischemic encephalopathy. Therapeutic hypothermia has been reported to reduce the incidence and severity of neonatal AKI, while perinatal asphyxia increases adverse outcomes, including death [24, 25].

Our study found a low incidence of AKI, and we believe that one of the limitations that explains these findings is the probability of underdiagnosis during the initial months of the SARS CoV-2 pandemic due to lower patient recruitment. Personnel at the included NICUs used a standard technique to weigh diapers and determine urine output when patients did not have a bladder catheter; this could introduce a measurement bias in the urine output. There was variability in the frequency of serum creatinine measurements depending on individualized management and minimum intervention protocols standardized in each of the NICUs. It was also difficult to adhere to the KDIGO classification when an increase of creatinine (0.3 mg/dl) occurred in relation to a very low previous value. Finally, when applying the KDIGO classification, we found it difficult to diagnose and classify AKI in patients with a persistently high creatinine value which did not meet the required 0.3 mg/dl increase. In that regard, some authors have considered the need to optimize criteria by including an increase in serum creatinine ≥0.1 mg/dl for newborns older than 29 weeks [26].

## Conclusions

Careful attention to serum creatinine and urine output is required in newborns of any gestational age admitted to the NICU, especially between 2 and 9 days of life, which is the period during which AKI more frequently develops. Kidney function recovery is higher, and mortality is lower in patients with AKI-stage 1 compared to those in a severe stage. Consequently, it is important to ensure adequate use of the KDIGO classification.

## Data Availability

The datasets generated and analyzed during the current study are available in the Science Data Bank repository, 10.11922/sciencedb.00896.

## Abbreviations

CLE: La Estancia Clinic
G: Grams
HUSJ: San José University Hospital
HSLV: Susana López de Valencia Hospital
CI: Confidence interval
AKI: Acute kidney injury
KDIGO: Kidney Disease: Improving Global Outcomes
Me: Median
IQR: Inter-quartile range
GFR: Glomerular filtration rate
RRT: Renal replacement therapy
NICUs: Neonatal intensive care units
VS: Versus
